# Nigerian physiotherapists’ knowledge, attitude, and practice of digital physical therapy: a cross-sectional study

**DOI:** 10.1186/s43161-022-00118-3

**Published:** 2023-02-28

**Authors:** Taofeek Oluwole Awotidebe, Olufesola Motunrayo Fasakin, Olufemi Oyeleye Oyewole, Usman Eniola Bello, Adekola Babatunde Ademoyegun, Ayodele Teslim Onigbinde, Chidozie E. Mbada, Adekola John Odunlade, Rufus Adesoji Adedoyin

**Affiliations:** 1 Department of Medical Rehabilitation, College of Health Sciences, Obafemi Awolowo University, Ile-Ife, Nigeria; 2 Nursing Services Department, Veterans Affairs Medical Center, 4500 South Lancaster Road, Dallas, TX 75216 USA; 3 Department of Physiotherapy, Olabisi Onabanjo University Teaching Hospital, Sagamu, Ogun State Nigeria; 4 Department of Physiotherapy, Federal Medical Centre, Idi – Aba, Abeokuta, Ogun State Nigeria; 5 Department of Physiotherapy, Osun State University Teaching Hospital, Osogbo, Osun State Nigeria; 6 Department of Health Professions, Faculty of Health, Psychology and Social Care, Manchester Metropolitan University, Manchester, UK

**Keywords:** Knowledge, Attitude, Practice, Digital physical therapy, Physiotherapist, Nigeria

## Abstract

**Background:**

The increasing demand for physical therapy services worldwide calls for innovations to be made to meet the challenges of service delivery. However, little is known about the knowledge, attitude, and practice (KAP) of digital physical therapy among Nigerian physiotherapists. Hence, this study aimed to evaluate the level of KAP of digital physical therapy among physiotherapists in Nigeria.

**Methods:**

This cross-sectional study purposively recruited 150 Nigerian physiotherapists. Copies of modified and validated questionnaires on the KAP of digital physical therapy from a previous study were sent to registered and licensed physiotherapists via electronic survey to assess the KAP of digital physical therapy. Descriptive and inferential statistics were used to analyze the data. Alpha level was set at *p* < 0.05.

**Results:**

The mean age of the respondents was 29.76 ± 6.95 years. Most participants (81.3%) have a knowledge level above average, while 18.7% have below average. Furthermore, most (76.0%) of the respondents had a positive attitude toward digital physical therapy. In addition, more than two-thirds, 69.3% uses digital physical therapy platforms for physical therapy practice. Knowledge, attitude, and practice were not significantly associated with sociodemographic characteristics (*p* > 0.05). Furthermore, there was no significant association between knowledge, attitude, and practice (*p* > 0.05).

**Conclusion:**

Many Nigerian physiotherapists demonstrated adequate knowledge, positive attitude, and good practice of digital physical therapy. However, knowledge, attitude, and practice of digital physical therapy were not influenced by sociodemographic characteristics.

**Supplementary Information:**

The online version contains supplementary material available at 10.1186/s43161-022-00118-3.

## Introduction

Digital physical therapy is a term that is being used in tandem with several other words such as tele-physiotherapy, e-physiotherapy, and others to refer to the application of information and communication technologies in the provision of physical therapy services to people remotely in their homes or other environments [1]. Such services include consultations, therapeutic intervention via live or prerecorded materials, monitoring of home programs, education, and group therapy for people with similar conditions [2]. It is an aspect of physical therapy that has been gaining tremendous growth in the last decade [3] and experienced an explosive increase in significance due to the global impact of the coronavirus disease 2019 and its subsequent affectation on daily living through mandatory social distancing and other protocols that were instituted to ensure safety [4]. Hence, at the height of the pandemic physiotherapists turned en masse to digital physical therapy platforms once more to ensure uninterrupted delivery of their services to patient populations who might otherwise suffer long-term consequences or disability from interruption of their management sessions [5, 6].

A factor that has also contributed to the growth of digital physical therapy is the increasing availability of digital technologies especially mobile devices and their applications which have quickly taken on the role of crucial support for clinical decision-making to ensure better health outcomes [7]. Many mobile applications can be used for several aspects of physical therapy, for example, there are applications that provide information on exercise purposes, dosage, and techniques, as well as information on exercises for prophylactic and rehabilitative purposes [7]. Among the many practices employed by physiotherapists to rehabilitate individuals, therapeutic exercise stands as one of the most useful in restoring an individual to function as well as preventing disability [8, 9]. The physiotherapist selects the most appropriate exercise program based on the underlying origins of impairments, activity limitation, and participation restriction [10]. Based on scientific evidence, physical rehabilitation approaches for the recovery of function may have multiple effects on many systems of the body during rehabilitation; for instance, during specific exercise intervention, the musculoskeletal system may help to increase joint range of motion and muscle strength with additional effects on the nervous system with improved proprioception and sensorimotor control [11, 12]. Reports from systematic reviews support the ability of digital physical therapy to deliver this multi-beneficial therapeutic exercise effectively with a moderate to high-quality difference when compared to other intervention mediums [13].

Evidence suggests that patient perspective on the usage of these digital tools for health care is mostly a positive experience; hence, many countries in the world have adopted digital physical therapy for use in the delivery of physical therapy services [14, 15]. Previously, Odole et al. [16] identified six themes responsible for perceived challenges to the practice of digital physical therapy by physiotherapists in Nigeria. These themes include “inadequate and underdeveloped infrastructure, ethical issues, training of physiotherapists/patients’ literacy needs, physiotherapists-patient contact, cultural issues, and financial implications.” However, the recent advances in the technical competencies of Nigerians as well as the impact of the coronavirus pandemic have caused a positive shift in the use of digital technologies for health care. Presently, there is a dearth of information on the current level of competency, attitude, and practice of digital physical therapy among Nigerian physiotherapists. This is necessary to provide a critical hint into the possible ways of speeding up the implementation of digital physical therapy practice on a national scale. Hence, the purpose of this study was to investigate the current level of knowledge, practice, and attitude toward the use of digital physical therapy among physiotherapists in Nigeria.

## Methods

This was a cross-sectional study. The respondents for this study were physiotherapists from selected government hospitals in southwest Nigeria using a purposive sampling technique. Eligibility for inclusion into the study was apparently healthy clinical physiotherapists licensed by the Medical Rehabilitation Therapists Board of Nigeria and clinical physiotherapists working in selected government hospitals/clinics in southwest Nigeria. Respondents were excluded from the study if they were physiotherapists who retired from active service and those that are practicing outside of southwest Nigeria. The sample size was determined using a standardized formula [17] with a 95% confidence level set at 1.96, the expected proportion in the population at 39%, and the absolute error/precision at 10% [18]. Thus, an estimated minimum sample size of ninety-one (91) respondents was expected to power the study but recruited 150 respondents for the study.

### Knowledge, attitude, and practice of digital physical therapy questionnaire

The instrument used in this study was an adapted but validated questionnaire from a previous study by Mbada et al. [18]. The questionnaire was originally designed to assess the awareness, attitude, and expectations of physiotherapy students on telerehabilitation. The questionnaire was modified, restructured, and pilot-tested in a sample of physiotherapists who were not the main part of this study. The questions that were specific to knowledge, attitude, and practice were retained. The modification includes changing the word “telerehabilitation” to digital physical therapy and “telemedicine” to telephysiotherapy. Debriefing of participants that participated in the pilot study revealed adequacy of content and face validity. The questionnaire had two sections; the first section collects sociodemographic data such as age, sex, and cadre. In addition to the sources of discovery of digital physical therapy by respondents, this first section also contained an item that assessed practice settings among respondents. The second section contained a total of twenty-six questions designed to evaluate the level of knowledge (6 items), attitudes (10 items), and practice (10 items) of respondents. The results from this section were scored on a 5-point Likert scale (strongly disagree, disagree, I do not know, agree, and strongly agree). “Strongly disagree,” “disagree,” and “I don’t know” were assigned a score of zero (0), while “agree” and “strongly agree” scored one (1). The maximum score for knowledge was six, ten for attitudes, and practice while the least is zero.

### Procedure

Ethical approval was obtained from the Health Research and Ethics Committee of the Institute of Public Health, College of Health Sciences, Obafemi Awolowo University Ile-Ife, with protocol number IPH/OAU/12/1678. The purpose of the study was explained to the respondents, and informed consent was sought from all respondents by text message requesting if they were willing to participate in the study. Copies of the questionnaires were distributed to respondents through e-platforms such as Telegram and WhatsApp group chats. The questionnaire was self-administered.

### Data analysis

Descriptive statistics of frequency, percentage, mean, and standard deviation were used to summarize the data. The association between knowledge, attitude, practice, and sociodemographic characteristics was evaluated with the chi-square test. The questionnaire used was an ordinal Likert scale, and this informed the choice of a nonparametric test. Statistical analysis was performed using the IBM Statistical Package of Social Sciences (SPSS) (Version 21). The alpha level was set at *p* < 0.05.

## Results

**Fig. 1:**
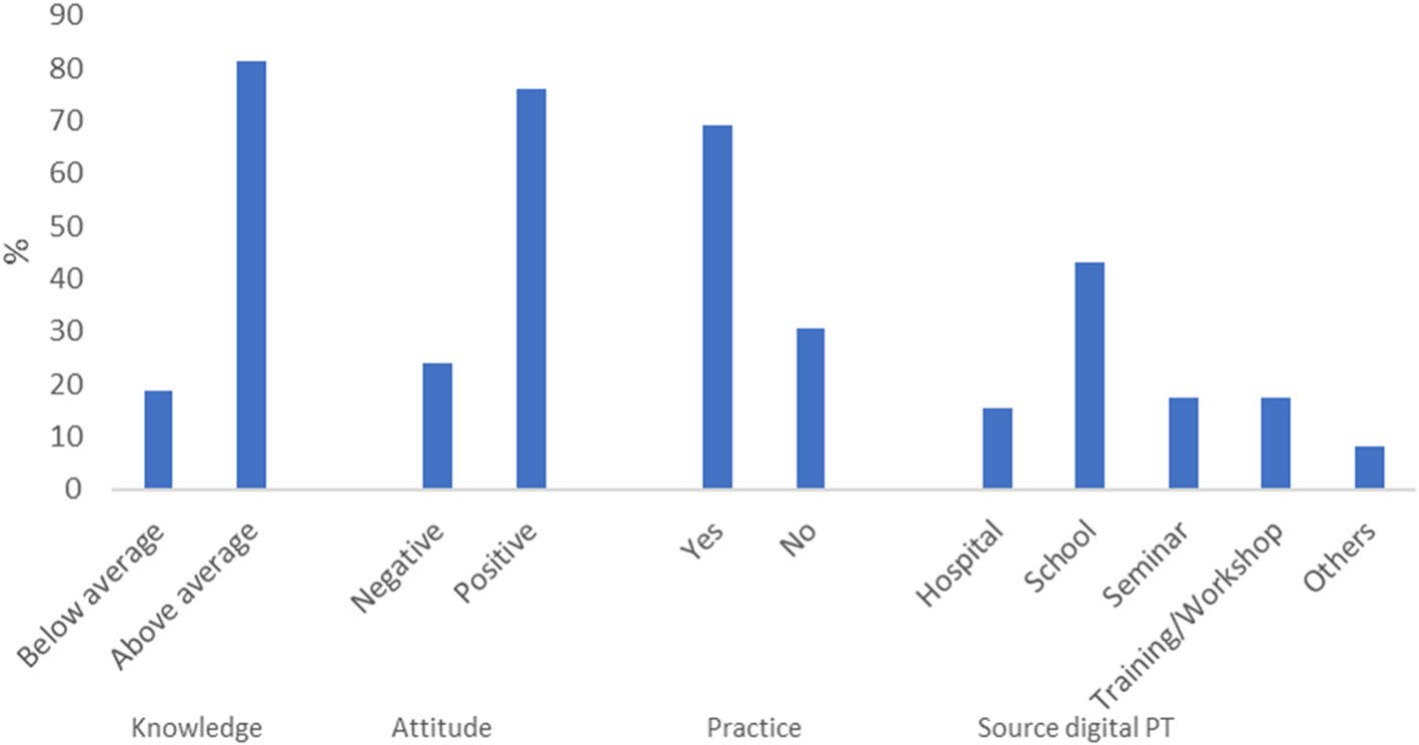
Distribution of knowledge, attitude, practice, and sources of information about digital physical therapy

Table 1 shows the sociodemographic data of respondents. The results reveal that most of the respondents were between the age groups of below 30 years with a mean age of 29.76 ± 6.95 years. Less than two-thirds, 60.7% of the respondents were male respondents, while more than a third, 35.3% and 34.70% were physiotherapy interns and physiotherapist cadre, respectively. Figure 1 shows the distribution of knowledge, attitude, practice scores, and main sources of information about digital physical therapy. The results show that most (81.3%) of respondents had above average knowledge of digital physical therapy, 76.0% reported a positive attitude, and 69.3% were involved in the regular practice of digital physical therapy. The main sources of information were schools, training/workshop, and hospital, 17.3%, 15.3%, and 8.0%, respectively. Furthermore, Table 2 illustrates the knowledge component of digital physical therapy. In a majority, 93.3% of respondents correctly identified that digital physical therapy refers to the use of ICT to provide physical therapy to people remotely in their homes. Similarly, most respondents (90.7%) asserted that digital physical therapy services include evaluations and means of education and networking for people with disabilities. Also, most respondents (86.0%) reported that digital physical therapy is the same as tele-physiotherapy.

**Table 1:** Sociodemographic data of respondents (*N* = 150)

Variable	Frequency	Percentage	Mean ± SD
Age (years)			29.76 ± 6.95
< 30	85	56.7	
30–40	55	36.7	
> 40	10	6.7	
Sex			
Male	91	60.7	
Female	59	39.3	
Cadre			
Intern PT	53	35.3	
PT	52	34.7	
Senior PT	19	12.7	
Chief PT	17	11.3	
Director cadre	9	6.0	

Key: *PT* physiotherapist, *SD* standard deviation

**Table 2:** Physiotherapists’ knowledge, practice of, and attitude toward digital physical therapy (*N* = 150)

Item	Disagree^a^ %	Agree^a^ %
Digital PT refers to the use of information and communication technologies (ICT) to provide rehabilitation services to people remotely in their home or other environments	4.70	93.30
Digital PT services include evaluation, therapeutic interventions, remote monitoring of progress, education, training, and a means of networking for people with disabilities	6.00	90.70
Digital PT is the same as tele-physiotherapy	7.30	76.00
Digital PT enables access to care for individuals in remote areas or for those who have mobility issues associated with physical impairment, access to transport, and socioeconomic factors	8.00	83.30
Adequate funding and policy for digital physical therapy systems in Nigeria are needed	4.60	92.00
I have issues with Internet devices such as smartphones, tablets, and computers as I am not able to use them	78.70	17.30
Digital PT is convenient as I may not have to leave my environment	8.60	84.70
Digital PT enables patient to take control of the management of their condition unlike the face-to-face in-person approach	23.30	62.00
I find it easy to learn and use digital PT systems	9.30	72.60
I believe I could be more productive quickly using digital PT	16.00	70.0
The way I interact with digital PT systems is satisfactory	18.70	56.7
I like using digital PT systems	12.70	59.3
Digital PT systems are able to do everything I would want them to do	48.00	28.0
Digital PT will benefit only the urban community	48.00	40.7
I presume patients would feel comfortable in being treated by digital PT	29.30	43.3
Digital PT can never replace face-to-face consultations	23.30	66.0
I will accept digital PT only after seeing reports of patients being treated by it	36.0	49.3
Digital PT should be implemented in all hospitals	10.7	72.7
Due to lack of sufficient knowledge of digital PT systems, I am unable to practice it	54.7	33.4
Due to the large number of patients in my practice, I am not interested in digital PT	72.0	13.3
Digital PT is a waste of my valuable time	78.6	6.0
Feedback should be sent after each session to aid my use of digital PT	6.0	83.4
Network availability in remote areas should be enhanced for digital PT to be functional	2.0	92.7
Patient-clinician acceptability of digital PT is needed or should be improved	2.0	90.0
Confidentiality, patient privacy, abuse of use by patients, Internet fraud, and quackery should be minimized to zero	4.7	87.4
I would recommend digital physical therapy to family and friends	6.7	75.3

^a^Some items may not sum up 100% due to omitted options

Table 2 shows the attitude toward digital physical therapy. Most respondents (92.0%) supported the statement that adequate funding and policy for digital physical therapy in Nigeria are needed. Similarly, a majority, 82.7% of respondents disavowed the notion that issues with Internet devices prevented them from using digital physical therapy. Furthermore, less than two-thirds (62.0%) of respondents affirmed that digital physical therapy may enable more patient-oriented health care. However, less than two-thirds (59.3%) of respondents reported that they liked using digital physical therapy systems. Interestingly, a majority, 70.0% of respondents believed they could be more productive using digital physical therapy.

Table 2 shows the respondents’ practice of digital physical therapy. More than two-thirds (69.30%) of the respondents use digital physical therapy platforms for their physical therapy practice. Similarly, two-thirds (66.0%) of respondents believed digital physical therapy could never replace face-to-face consultations. Contrarily, less than half, 49.3% of respondents reported that they could only accept digital physical therapy after seeing reports of patients being treated by it. Nonetheless, a majority, 72.7% of respondents support the notion that digital physical therapy should be implemented in all hospitals.

Table 3 shows the associations between knowledge level, attitude, the practice of digital physical therapy, and the sociodemographic characteristics of respondents. The results showed there was no significant association between knowledge levels and sociodemographic characteristics (*p* > 0.05). Table 4 shows the associations between knowledge level, attitude, and practice of digital physical therapy. The results show no significant association between knowledge and each of the attitude to practice status of respondents (*p* > 0.05).

**Table 3:** Test of associations between knowledge, attitude, practice of digital physical therapy, and sociodemographic characteristics of respondents

	Knowledge				Attitude				Practice			
	Below average	Above average			Negative	Positive			No	Yes		
Age group (years)	n(%)	n(%)	χ^2^	p-value	n(%)	n(%)	χ^2^	p-value	n(%)	n(%)	χ^2^	p-value
<30	12 (8.0)	59 (39.0)	7.032	0.134	20 (13.3)	65 (43.3)	0.162	0.922	30 (20.0)	55 (36.7)	2.094	0.351
30-40	10 (6.7)	34 (22.7)			14 (9.3)	41 (27.3)			14 (9.3)	41 (27.3)		
>40	6 (4.0)	29 (19.3)			8 (5.3)	2 (0.0)			2 (1.3)	8 (5.3)		
Sex												
Male	32 (21.3)	43 (28.7)	0.238	0.888	20 (13.3)	71 (47.3)	0.519	0.471	24 (16.0)	67 (44.7)	2.005	0.154
Female	19 (12.7)	28 (18.7)			16 (10.7)	43 (28.7)			22 (14.7)	37 (24.7)		
Cadre												
Intern PT	21 (14.0)	21 (14.0)	6.321	0.611	14 (9.3)	39 (26.0)	3.397	0.494	20 (13.3)	33 (22.0)	7.017	0.135
PT	17 (11.3)	24 (16.0)			14 (9.3)	38 (25.3)			15 (10.0)	37 (24.7)		
Senior PT	8 (5.3)	9 (6.0)			2 (1.4)	17 (11.3)			4 (2.7)	15 (10.0)		
Chief PT	3 (2.0)	11 (7.3)			5 (3.3)	12 (8.0)			7 (4.7)	10 (6.7)		
Director	2 (1.4)	6 (4.0)			1 (0.7)	8 (5.3)			0 (0.0)	9 (6.0)		

Key: *PT* physiotherapist

**Table 4:** Test of associations between knowledge, attitude, and practice of digital physical therapy

Knowledge				
Variable	Below average	Above average		
	*n* (%)	*n* (%)	*χ*^2^	*p*-value
Attitude				
Negative	10 (6.7)	26 (17.3)	0.220	1.504
Positive	18 (12.0)	96 (64.0)		
Practice				
No	13 (8.7)	33 (22.0)	0.091	4.793
Yes	15 (10.0)	89 (59.0)		

## Discussion

The objectives of this study were to evaluate the knowledge, attitude, and practice of digital physical therapy among Nigerian physiotherapists. Furthermore, the associations between knowledge, attitude, and practice of digital physical therapy and the sociodemographic characteristics of respondents were also explored. Most participants have adequate knowledge, a positive attitude, and practice digital physical therapy. These were not associated with demographic variables nor were the knowledge, attitude, and practice of digital physical therapy associated with each other. Findings from this study showed that respondents were mostly young adults. Similarly, most respondents in this study were young physiotherapy interns and physiotherapist cadre. This finding is similar to previous studies reporting that social media platforms attract more of younger populations known as digital natives and some older populations known as digital immigrants [18, 19]. This could be attributed to the ability of the younger generation to easily adapt to any form of technology, especially information and communication technology (ICT). This appears to be trending worldwide as younger generations of all strata of life are more comfortable with the use of ICT, and they find it easy to have a better understanding than the older generations.

In the present study, many of the physiotherapists who participated in this study use digital physical therapy platforms for physical therapy practices, and they got to know about these platforms mostly through schools, seminars, training, and workshops. This finding contradicts a previous submission that professional training is a barrier to digital physical therapy [20]. However, it should be noted that the previous study was published more than a decade ago, and the scheme of education might have improved since then. The relatively young professionals in this study displayed adequate knowledge, positive attitude, and practice of digital physical therapy, which is also consistent with previous studies on digital healthcare adoption among younger populations [21]. This finding could provide a crucial hint into the possible ways of speeding up the implementation of digital physical therapy practice on a national scale [22].

It is noteworthy that access to healthcare services is a big challenge in Nigeria [23]. However, findings from this study show that most of the respondents correctly identified that digital physical therapy enables access to care for individuals with mobility issues associated with physical impairment, access to transport, and socioeconomic factors. Furthermore, a majority also confirm that they could recommend digital physical therapy to family and friends. This finding is similar to a previous study in that physiotherapists are willing to recommend and use digital physical therapy for their clients [24]. This shows that most respondents also have adequate knowledge and a positive attitude toward digital physical therapy. However, some other items in the survey reveal some potent skepticism on the part of the respondents toward digital physical therapy, for instance, only a few numbers of respondents believed patients would feel comfortable being treated by digital physical therapy, and some also believed in the ability of digital physical therapy systems to do everything they would want them to do. The disparity in knowledge may be due to the level of exposure to ICT among cadres of physiotherapists in this study.

Information and communications technology infrastructure is a prerequisite for digital healthcare implementation. Since ICTs are composite measures of Internet and mobile applications, ICT usage for digital health care must embrace not only mobile network coverage or mobile phone subscriptions but also Internet technologies and web applications [2, 25]. Furthermore, the World Confederation of Physiotherapists [26] reported that successful and secure digital consultations depend on the integrity of the key technology platforms. An alternative communication pathway may be required where Internet connectivity is inadequate, while in some circumstances, poor or absent Internet connectivity may impact the ability to deploy digital practice options. However, this study’s results reveal that most respondents are skeptical that issues with Internet devices prevent them from using digital physical therapy.

Findings from this study show that most respondents assert that large numbers of patients do not discourage them from practicing digital physical therapy. They also reject the notion that digital physical therapy wastes their valuable time. Likewise, a previous study opined that physiotherapists are favorably disposed to the adoption and practice of digital physical therapy [27]. However, other potential points of skepticism arise from issues of ethics, lack of confidentiality, abuse by patients, Internet fraud, and quackery with most respondents also supporting the movement that these issues need to be minimized to zero, which reveals skepticism in the level of trust of digital physical therapy platforms by respondents for this study. Hence, policymakers in the health sector need to pay more attention to ICT trends to be able to communicate correct information and policies designed [15, 28]. This will help to cater to salient issues and ultimately curtail the skepticism before they become significant enough that may hinder the advance of digital physical therapy.

The findings of this study have some policy implications. With the inadequate physiotherapy-to-patient ratio in Nigeria, the practice of digital physical therapy should be encouraged and advocated for in all hospitals. Since most physiotherapists have a positive attitude toward the practice of digital physical therapy, policymakers should facilitate progressive and pragmatic policies to ensure the seamless integration of digital physical therapy in Nigeria. These will not only reduce waiting time but provide services to those in remote areas and thereby may achieve universal health coverage in Nigeria. The current digital physical therapy curriculum should be revised and updated to keep up with the latest innovations. Feedback after each session should be encouraged to identify ways of improving the systems. Adequate funding for digital physical therapy systems and research needs to be improved.

It is important to identify the potential limitations of this study. This study design is cross-sectional, and issues of self-reported information, which might be affected by reporting bias, must be considered when determining the interpretation and generalizability of this study. However, the strength of this study lies in the heterogeneity of the place of work of physiotherapists that participated in the study, thus limiting the extent of bias. Future studies should explore mixed methods design to investigate knowledge, attitude, and practice of digital physical therapy to provide more in-depth information about the subject. Acceptability of digital physical therapy among physiotherapy service users in Nigeria is warranted.

## Conclusion

Most physiotherapists have adequate knowledge and a positive attitude toward digital physical therapy. Most physiotherapists also use digital physical therapy platforms for their physical therapy practice. However, knowledge, attitude, and practice of digital physical therapy were not influenced by sociodemographic characteristics.

## Supplementary Information

**Additional file 1: Appendix 1.** Knowledge, Attitude, and Practice of Digital Physical Therapy Questionnaire.

## Data Availability

The datasets used and/or analyzed during the current study are available from the corresponding author on reasonable request.

## Abbreviations

KAP: Knowledge, attitude, and practice
ICT: Information and communication technology
