# Supplementary material for: Nigerian physiotherapists’ knowledge, attitude, and practice of digital physical therapy: a cross-sectional study
Source: Bull Fac Phys Ther. 2023 Feb 28;28(1):5. doi: 10.1186/s43161-022-00118-3 (PMC9970850; doi:10.1186/s43161-022-00118-3)
Supplement: Supplementary file 1 — Additional file 1: Appendix 1. Knowledge, Attitude, and Practice of Digital Physical Therapy Questionnaire. [file 43161_2022_118_MOESM1_ESM.docx]

**APPENDIX 1**

**Knowledge, Attitude, and Practice of Digital Physical Therapy Questionnaire**

This questionnaire is designed to assess knowledge, attitude, and practice of physiotherapists towards digital physical therapy. The purpose of this research is to help understand and reposition digital physical therapy as a platform of care in physiotherapy. It will be appreciated that you fill the questionnaire with all sincerity. You are assured that your response will be kept confidential.

Thank you for your anticipated cooperation

**Section A- Sociodemographic data**

Age ……………………………………

Sex: Male ( ) Female ( )

Cadre: Intern ( ) Physiotherapists ( ) Senior Physiotherapists ( )

Chief Physiotherapists ( ) Assistant Director ( ) Director ( )

Do you use digital physical therapy platforms such as OTS videoconferencing, PT Pal Pro, eHAB and others in your physical therapy practice?

Yes ( ) No ( )

How did you get to know about digital physical therapy?

School ( ) Hospital ( ) Workshop( ) Seminar ( )

Training ( ) Others ( )

**Section B**

For each of the following statements, please indicate your opinion on a five-point scale: Strongly Disagree (SD), Disagree(D), I don’t know (IDK), Agree(A), Strongly Agree (SA). Tick the response/number that correspond with your answer. There are no correct or wrong answer, only your opinions.

NB: Digital physical therapy is abbreviated as Digital PT

| **S/N** | **Question** | **Strongly**  **disagree** | **Disagree** | **I don’t know** | **Agree** | **Strongly agree** |
| --- | --- | --- | --- | --- | --- | --- |
| 1. | Digital PT refers to the use of information and communication technologies (ICT) to provide rehabilitation services to people remotely in their home or other environments |  |  |  |  |  |
| 2. | Digital PT services include evaluation, therapeutic interventions, remote monitoring of progress, education, training and a means of networking for people with disabilities |  |  |  |  |  |
| 3. | Digital PT is the same as telephysiotherapy |  |  |  |  |  |
| 4. | Adequate funding and policy for digital physical therapy systems in Nigeria is needed |  |  |  |  |  |
| 5. | I have issues with internet devices such as smartphones, tablets, and computers as I am not able to use them. |  |  |  |  |  |
| 6. | Digital PT is convenient as I may not have to leave my environment. |  |  |  |  |  |
| 7. | Digital PT enables patient to take control of the management of their condition unlike the face-to-face in person approach |  |  |  |  |  |
| 8. | Digital PT enables access to care for individuals in remote areas or for those who have mobility issues associated with physical impairment, access to transport and socioeconomic factors |  |  |  |  |  |
| 9. | I find it easy to learn and use digital PT systems |  |  |  |  |  |
| 10. | I believe I could be more productive quickly using digital PT |  |  |  |  |  |
| 11. | The way I interact with digital PT systems is satisfactory |  |  |  |  |  |
| 12. | I like using Digital PT systems |  |  |  |  |  |
| 13. | Digital PT systems are able to do everything I would want them to do |  |  |  |  |  |
| 14. | Digital PT will benefit only the urban community |  |  |  |  |  |
| 15. | I presume patients would feel comfortable in being treated by digital PT. |  |  |  |  |  |
| 16. | Digital PT can never replace face-to-face consultations |  |  |  |  |  |
| 17. | I will accept digital PT only after seeing reports of patients being treated by it |  |  |  |  |  |
| 18. | Digital PT should be implemented in all hospitals |  |  |  |  |  |
| 19. | Due to lack of sufficient knowledge of digital PT systems I am unable to practice it |  |  |  |  |  |
| 20. | Due to the large number of patients in my practice, I am not interested in digital PT |  |  |  |  |  |
| 21. | Digital PT is a waste of my valuable time |  |  |  |  |  |
| 22. | Feedback should be sent after each session to aid my use of digital PT |  |  |  |  |  |
| 23. | Network availability in remote areas should be enhanced for digital PT to be functional |  |  |  |  |  |
| 24. | Patient-clinician acceptability of digital PT is needed or should be improved |  |  |  |  |  |
| 25. | Confidentiality, patient privacy, abuse of use by patients, internet fraud and quackery should be minimized to zero. |  |  |  |  |  |
| 26. | I would recommend digital physical therapy to family and friends |  |  |  |  |  |

Thank you for your participation.
